# Genome-wide identification and expression analysis of the GRAS transcription in eggplant (*Solanum melongena* L.)

**DOI:** 10.3389/fgene.2022.932731

**Published:** 2022-09-02

**Authors:** Ting Yang, Cheng Li, Hui Zhang, Jingyu Wang, Xiaofang Xie, Yongxian Wen

**Affiliations:** ^1^ Fujian Key Laboratory of Crop Breeding By Design, Fujian Agriculture and Forestry University, Fuzhou, China; ^2^ Institute of Statistics and Applications, Fujian Agriculture and Forestry University, Fuzhou, China; ^3^ College of Life Sciences, Fujian Agriculture & Forestry University, Fuzhou, China; ^4^ College of Computer and Information Science, Fujian Agriculture and Forestry University, Fuzhou, China

**Keywords:** eggplant, GRAS, phylogenetic analysis, expression pattern, cold stress

## Abstract

GRAS proteins are plant-specific transcription factors and play important roles in plant growth, development, and stress responses. In this study, a total of 48 *GRAS* genes in the eggplant (*S. melongena*) genome were identified. These genes were distributed on 11 chromosomes unevenly, with amino acid lengths ranging from 417 to 841 aa. A total of 48 GRAS proteins were divided into 13 subgroups based on the maximum likelihood (ML) model. The gene structure showed that 60.42% (29/48) of *SmGRAS*s did not contain any introns. Nine pairs of *SmGRAS* appeared to have a collinear relationship, and all of them belonged to segmental duplication. Four types of cis-acting elements, namely, light response, growth and development, hormone response, and stress response, were identified by a cis-acting element predictive analysis. The expression pattern analysis based on the RNA-seq data of eggplant indicated that *SmGRAS*s were expressed differently in various tissues and responded specifically to cold stress. In addition, five out of ten selected *SmGRAS*s (*SmGRAS2/28/32/41/44*) were upregulated under cold stress. These results provided a theoretical basis for further functional study of *GRAS* genes in eggplant.

## Introduction

The name of the *GRAS* gene family is derived from its first three identified members, namely, gibberellic acid insensitive (GAI) (Peng et al., 1997), repressor of GA1 (RGA) (Silverstone et al., 1998), and scarecrow (SCR) (Di Laurenzio et al., 1996). Members of the GRAS protein family have great differences in sequence length and structure. Typical GRAS proteins are generally composed of 400–770 amino acids, including a variable N-terminal sequence and a relatively conserved C-terminal sequence (Bolle, 2004). The conserved C-terminal of typical GRAS has five different sequence motifs: leucine heptad repeat I (LHRI), VHIID, leucine heptad repeat II (LHRII), PFYRE, and SAW (Pysh et al., 1999). It was reported that GRAS proteins of rice, *Arabidopsis*, *Populus*, grape, and tomato were divided into 13 subfamilies: DELLA, HAM, LISCL, AtSCR, AtPAT1, DLT, AtSCL3, AtSCL4/7, AtLAS, AtSHR, Pt20, Os19, and Os4 based on phylogenetic analysis (Liu and Widmer, 2014).

Many studies have confirmed that GRAS proteins are important proteins in plant growth and development. For example, *AtLAS* controls the formation of the axillary meristem (Greb et al., 2003). *OsSCR* regulates asymmetric cell division (Kamiya et al., 2003). *AtSCL3* is a tissue-specific integration factor of the GA pathway, which can promote the division and elongation of *Arabidopsis* root cells (Heo et al., 2011). In addition, *GRAS* genes are also involved in plant responses to various stresses. *AtSCL14* interacts with Class II TGA to activate the detoxification system of the plant to reduce harm (Fode et al., 2008). In *Arabidopsis thaliana*, overexpression of *BnLAS* in *Brassica napus* leads to smaller stomatal opening, more wax deposition in leaves, and a lower water loss rate, indicating that *BnLAS* has potential applications in improving drought tolerance of plants (Yang, et al., 2011). In tomatoes, the transcript accumulation of *SlGRAS4* exhibited more than 250-fold change during cold stress compared to that in the control plants, which means that GRAS can respond positively to cold stress (Huang et al., 2015). In grapes, overexpression of *VaPAT1* gene, a member of the PAT1 subfamily, significantly increased plant resistance to cold by regulating jasmonic acid biosynthesis (Yuan et al., 2016; Wang et al., 2021). Torres-Galea et al. (2006) detected *AtSCL13* expression patterns in PAT1 branches of *Arabidopsis thaliana* and found that *AtSCL13* expression was induced under low temperature stress.

Eggplant (*Solanum melongena* L.) is a popular vegetable. It is cultivated all over the world, with the largest acreage in Asia. Eggplant is a kind of temperature-bias plant (Saini and Kaushik, 2019), and it is much more sensitive to low temperatures than other solanaceous vegetables (Wan et al., 2014), which is one of the main factors affecting the production of eggplant.

There are few studies on genes related to low-temperature tolerance in eggplants, which mainly focus on the analysis of genes related to low-temperature tolerance in *A. thaliana* and the mining of low-temperature tolerant genes in wild eggplant based on high-throughput sequencing technology. Wan et al. (2014) transferred *AtCOR15* and *AtCBF3* into the eggplant cultivar Sanyueqie and assessed their cold tolerance. The results showed that the expression of the exogenous *AtCBF3* and *AtCOR15A* could promote the cold adaptation process to protect eggplant plants from chilling stress (Wan et al., 2014). Zhou et al. (2020) isolated an ICE1-like gene (*SmICE1a*) from eggplant and functionally characterized its role in cold tolerance by overexpressing it in *A. thaliana*. The findings of this study indicated that the *SmICE1a* gene can be used to enhance cold tolerance in eggplant. A study using high-throughput sequencing technology to study the miRNA and its target genes of *Solanum aculeatissimum* at low temperatures obtained and verified nine significantly differentially expressed miRNAs and 12 targeted mRNAs (Yang et al., 2017). Therefore, excavating key cold resistance genes of eggplant is of significance for the cold resistance varieties’ breeding. At present, the response mode of GRAS transcription factor family members of eggplant under low temperature stress is not clear.

In this study, a comprehensive investigation of the *GRAS* gene family, including physicochemical properties, gene structure, conserved motifs, phylogeny, chromosome location, collinearity, cis-acting elements, and gene expression patterns under low temperature stress, was performed based on the current eggplant genome sequence data. The information derived from this study would provide a solid foundation for the further functional investigation of *GRAS* genes in eggplant.

## Materials and methods

### Screening and domain identification of eggplant GRAS proteins

The protein sequences of *A. thaliana* 33) and *Oryza sativa* L. (50) GRAS members (Liu and Widmer, 2014) were downloaded from TAIR (http://www.Arabidopsis.org/) and Phytozome (http://www.phytozome.net/search.php). These protein sequences were used as the queries to identify the GRAS orthologs in eggplant using the BLASTP tool of SpudDB and the Eggplant Genome Database (http://eggplant-hq.cn). Proteins with more than 30% similarity to the query sequence and an E-value less than E^−10^ were selected. The domains for GRAS proteins were further confirmed using the Conserved Domain Database (CDD) of NCBI (https://www.ncbi.nlm.nih.gov/Structure/bwrpsb/bwrpsb.cgi) and visualized by TBtools (Chen et al., 2020). The genes containing the GRAS domain were selected as the final candidates for eggplant *GRAS* genes and were renamed based on their physical position in the eggplant genome. The information of these genes was extracted from the eggplant database (http://eggplant-hq.cn), including gene IDs, physical position, gene sequence, and protein sequence. The molecular weight (MW), theoretical isoelectric point (pI), and protein length (PL) for the predicted SmGRAS proteins were calculated using the ExPASy online tool (http://prosite.expasy.org/) (Gasteiger et al., 2003).

### Gene structure and conserved motif analysis of eggplant GRAS proteins

The *SmGRAS* gene structures were identified using the Gene Structure Display Server (http://gsds.cbi.pku.edu.cn) (Guo et al., 2007). The conserved motifs were identified using MEME software (http://meme-suite.org) (Bailey et al., 2009). The parameters were set at 10 motifs with an optimum motif width of 50–300 residues. The conserved motifs were further annotated with the CDD tool (Marchler et al., 2017).

### Phylogenetic analysis of GRAS proteins

The GRAS protein sequences of eggplant, *Arabidopsis*, and rice were aligned using the multiple sequence alignment tool ClustalX (Thompson et al., 1997). The phylogenetic tree of GRAS family proteins was generated using the MEGA-X maximum likelihood (ML) model (Kumar et al., 2018) with 1,000 bootstrap replicates. The SmGRAS*s* were categorized based on taxonomic rules for the subfamily of *Arabidopsis* and rice GRAS protein sequences (Liu and Widmer, 2014).

### Chromosomal location and collinearity analysis of *SmGRASs*

According to the annotation information of the eggplant genome downloaded from the Eggplant Genome Database, the physical positions of *SmGRASs* on chromosomes were obtained, and the chromosome mapping was performed using TBtools software (Chen et al., 2020). Collinearity of *SmGRASs* was analyzed using MCScanX software (Wang et al., 2012), and Circos (Krzywinski et al., 2009) software was used for visualization. To identify gene duplication, the parameters for the proportion of alignment and the similarity of the two sequences were set to be greater than 70%. Two duplicated genes with a distance of less than 100 kb on the same chromosome were defined as tandem duplicates (Yang et al., 2020), while any other duplicated gene pairs that did not meet the parameters of tandem duplicated genes were all defined as segmental duplicated genes.

### Cis-acting element analysis of *SmGRASs*

According to the annotation information of the eggplant genome sequence, the 2 kb upstream sequence of *SmGRASs* was submitted to the PlantCARE website (http://bioinformatics.psb.ugent.be/webtools/plantcare/html) (Lescot et al., 2002) for cis-acting element prediction, and TBtools software (Chen et al., 2020) was used for visualization.

### Expression pattern analysis of *SmGRAS*s

According to the transcriptome data of eggplant (PRJNA328564 and PRJNA572318) published by NCBI, the FPKM (fragments per kilobase per million) value (log2 conversion) was used to analyze the expression of *GRAS* family genes under different tissues and low-temperature stress. Heatmaps of *SmGRAS* expression patterns were generated using the TBtools’ Heatmap module (Chen et al., 2020).

### Plant treatments and qRT-PCR analysis

The commercial species “Selected Purple Long Eggplant” was used for qRT-PCR. Eighteen plants were grown in a growth chamber at 28°C with a 16/8 h light/dark photoperiod until the fourth leaves were fully expanded. Then, nine plants of these seedlings were transferred to 4°C for 12 h. The seedlings grown under normal conditions were used as a control. The treated and control plantlets were collected 12 h after treatment and then stored at −80°C before RNA extraction.

The total RNA of the plantlets was extracted using TRIzol reagent (Invitrogen) according to the manufacturer’s instructions. The cDNA samples were then assessed by qRT-PCR using SYBR Premix ExTaq (Takara). Actin was used as an internal control gene. Three biological replicates (each containing three plants) and three technical replicates were measured for each treatment. The relative expression level of a gene was calculated according to the 2^−ΔΔCt^ method (Livak and Schmittgen, 2001). The primers used for qRT-PCR analysis are listed in Supplementary Table S1.

## Results and analysis

### Identification and physicochemical property analysis of the SmGRASs

A total of 48 SmGRASs containing GRAS domains were identified from the whole genome of eggplant and renamed from *SmGRAS1* to *SmGRAS48* based on their physical position on chromosomes (Table 1). The protein lengths of the 48 SmGRASs varied from 417 (SmGRAS6) to 841 (SmGRAS27) amino acids. The molecular weights ranged from 46,955.22 Da (SmGRAS6) to 95,884.91 Da (SmGRAS27). The theoretical isoelectric points (pI) of these SmGRASs varied from 4.95 (SmGRAS45) to 8.43 (SmGRAS34).

**TABLE 1:** *GRAS* genes identified in eggplant.

No.	Gene name	Gene id	Location (bp)	Chr	MW (Da)	pI	PL (aa)
1	*SmGRAS1*	*Smechr0100956.1*	9096627..9597	1	74440.13	5.83	657
2	*SmGRAS2*	*Smechr0100957.1*	9100908..9588	1	74295.13	6.03	656
3	*SmGRAS3*	*Smechr0101655.1*	16353704..16356949	1	82853.66	5.64	766
4	*SmGRAS4*	*Smechr0102147.1*	23751511..23085	1	59071.26	6.58	525
5	*SmGRAS5*	*Smechr0102148.1*	23755015..23177	1	68002.17	5.1	598
6	*SmGRAS6*	*Smechr0102782.1*	44313493..44868	1	46955.22	6.29	417
7	*SmGRAS7*	*Smechr0103899.1*	101543583..101983	1	52423.91	6.2	467
8	*SmGRAS8*	*Smechr0202369.1*	68167819..68195	2	67484.03	5.04	616
9	*SmGRAS9*	*Smechr0202394.1*	68366257..68555	2	60491.63	5.58	548
10	*SmGRAS10*	*Smechr0202592.1*	70117709..70951	2	58773.7	5.74	518
11	*SmGRAS11*	*Smechr0202609.1*	70298129..70059	2	47501.12	5.46	421
12	*SmGRAS12*	*Smechr0203187.1*	74994635..74828	2	76998.66	5.25	697
13	*SmGRAS13*	*Smechr0302081.1*	80300515..80024	3	71252.27	6.26	652
14	*SmGRAS14*	*Smechr0302579.1*	86165898..86167872	3	58899.44	6.61	528
15	*SmGRAS15*	*Smechr0303662.1*	95960868..95962490	3	60658.13	5.41	541
16	*SmGRAS16*	*Smechr0401109.1*	48573914..48575963	4	55008.66	5.36	495
17	*SmGRAS17*	*Smechr0401258.1*	55717340..55718779	4	53181.96	5.96	465
18	*SmGRAS18*	*Smechr0401532.1*	64833269..64836967	4	62884.01	5.11	561
19	*SmGRAS19*	*Smechr0500353.1*	3950772..3952937	5	80739.03	5.69	720
20	*SmGRAS20*	*Smechr0500354.1*	3953971..3958015	5	81748.31	5.32	726
21	*SmGRAS21*	*Smechr0500468.1*	5367175..5374708	5	60210.61	5.49	541
22	*SmGRAS22*	*Smechr0501999.1*	71422121..71424489	5	63678.71	6.42	567
23	*SmGRAS23*	*Smechr0502680.1*	80486152..80487825	5	57594.82	5.44	501
24	*SmGRAS24*	*Smechr0502748.1*	81063283..81065782	5	52495.18	6.09	469
25	*SmGRAS25*	*Smechr0600059.1*	897922..900511	6	65616.54	5.6	579
26	*SmGRAS26*	*Smechr0601319.1*	66235763..66237871	6	65621.08	6.38	599
27	*SmGRAS27*	*Smechr0602914.1*	87323086..87326478	6	95884.91	5.88	841
28	*SmGRAS28*	*Smechr0603017.1*	88208662..88210722	6	72410.31	6.1	636
29	*SmGRAS29*	*Smechr0701498.1*	84937623..84938921	7	48011.8	5.25	433
30	*SmGRAS30*	*Smechr0701743.1*	92623237..92627075	7	60658.9	6.36	546
31	*SmGRAS31*	*Smechr0701943.1*	97245321..97247331	7	49218.94	5.53	434
32	*SmGRAS32*	*Smechr0702503.1*	104537045..104540305	7	64262.94	6.27	578
33	*SmGRAS33*	*Smechr0702636.1*	105493514..105496196	7	59959.92	5.27	538
34	*SmGRAS34*	*Smechr0702726.1*	106308917..106310185	7	47913.35	8.43	423
35	*SmGRAS35*	*Smechr0800451.1*	6768043..6770159	8	51558.29	5.45	450
36	*SmGRAS36*	*Smechr0802185.1*	82575983..82579091	8	76017.79	5.74	701
37	*SmGRAS37*	*Smechr0802313.1*	84095305..84098713	8	75327.93	5.71	680
38	*SmGRAS38*	*Smechr0900438.1*	6411669..6415271	9	86289.75	5.75	764
39	*SmGRAS39*	*Smechr0900855.1*	18793688..18795304	9	55612.27	5.08	484
40	*SmGRAS40*	*Smechr0901929.1*	79662612..79664345	9	55711.21	6.23	500
41	*SmGRAS41*	*Smechr1000504.1*	6691939..6694766	10	62131.32	5.73	556
42	*SmGRAS42*	*Smechr1001636.1*	64747643..64751256	10	86484.02	5.73	760
43	*SmGRAS43*	*Smechr1001829.1*	68156739..68160660	10	89747.97	5.92	826
44	*SmGRAS44*	*Smechr1100032.1*	470509..473623	11	59993	5.72	540
45	*SmGRAS45*	*Smechr1100163.1*	1902184..1903926	11	63608.38	4.95	581
46	*SmGRAS46*	*Smechr1100616.1*	8037435..8039120	11	55463.21	5.19	489
47	*SmGRAS47*	*Smechr1102566.1*	98372183..98374214	11	60852.15	6.65	546
48	*SmGRAS48*	*Smechr1102664.1*	99602433..99606100	11	83061.76	5.69	738

### Gene structure and conserved motif analysis of *SmGRAS*s

**FIGURE 1:**
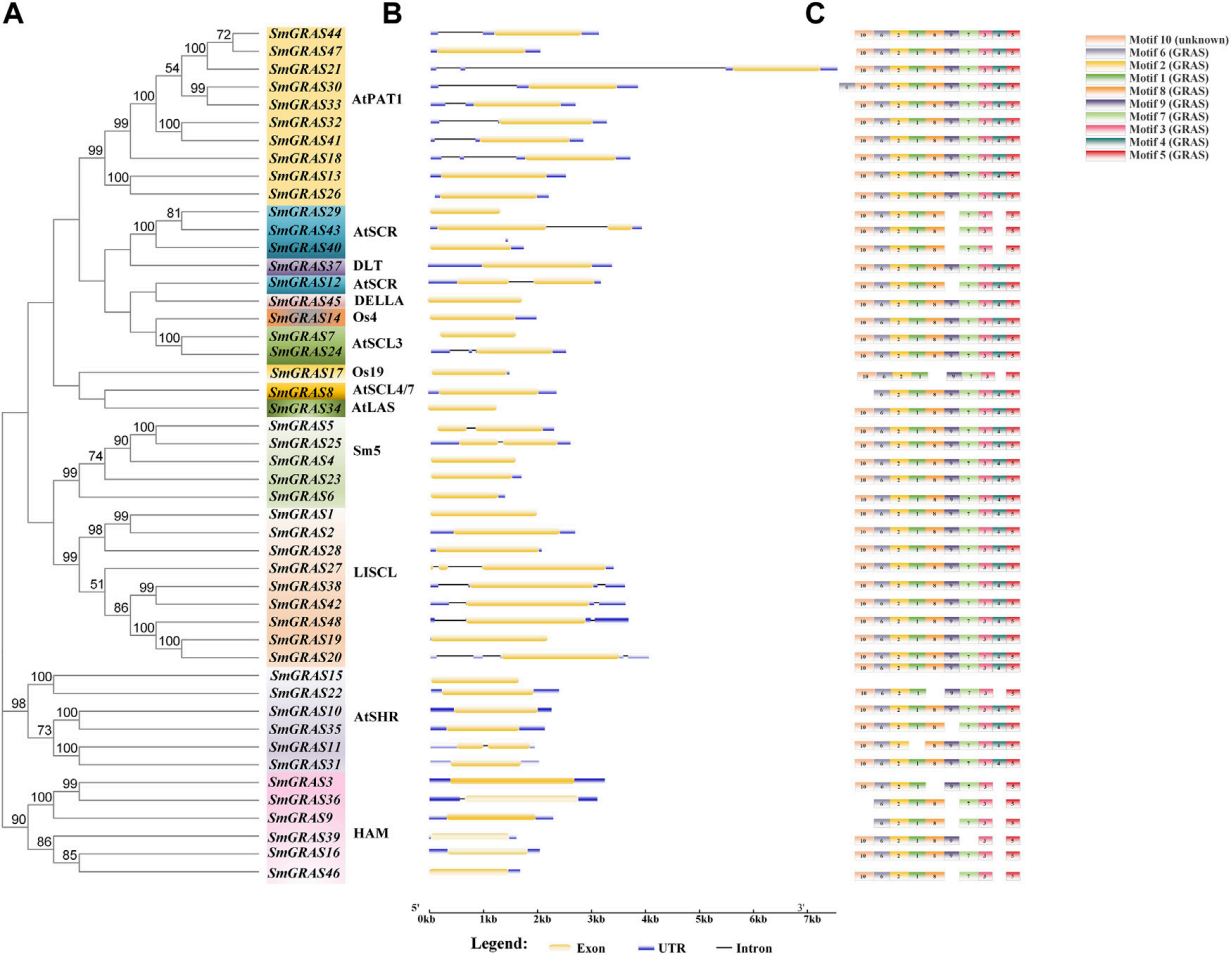
Phylogenetic tree, gene structure, and conserved motif of 48 GRAS transcription factors in eggplant. **(A)** Maximum likelihood (ML) tree generated by MEGA-X under the LG + G model with bootstrapping analysis (1,000 replicates). **(B)** Exon–intron structures of *SmGRASs* generated by the online software GSDS. **(C)** Conserved motifs of *SmGRASs* were further annotated with the CDD tool.

**FIGURE 2:**
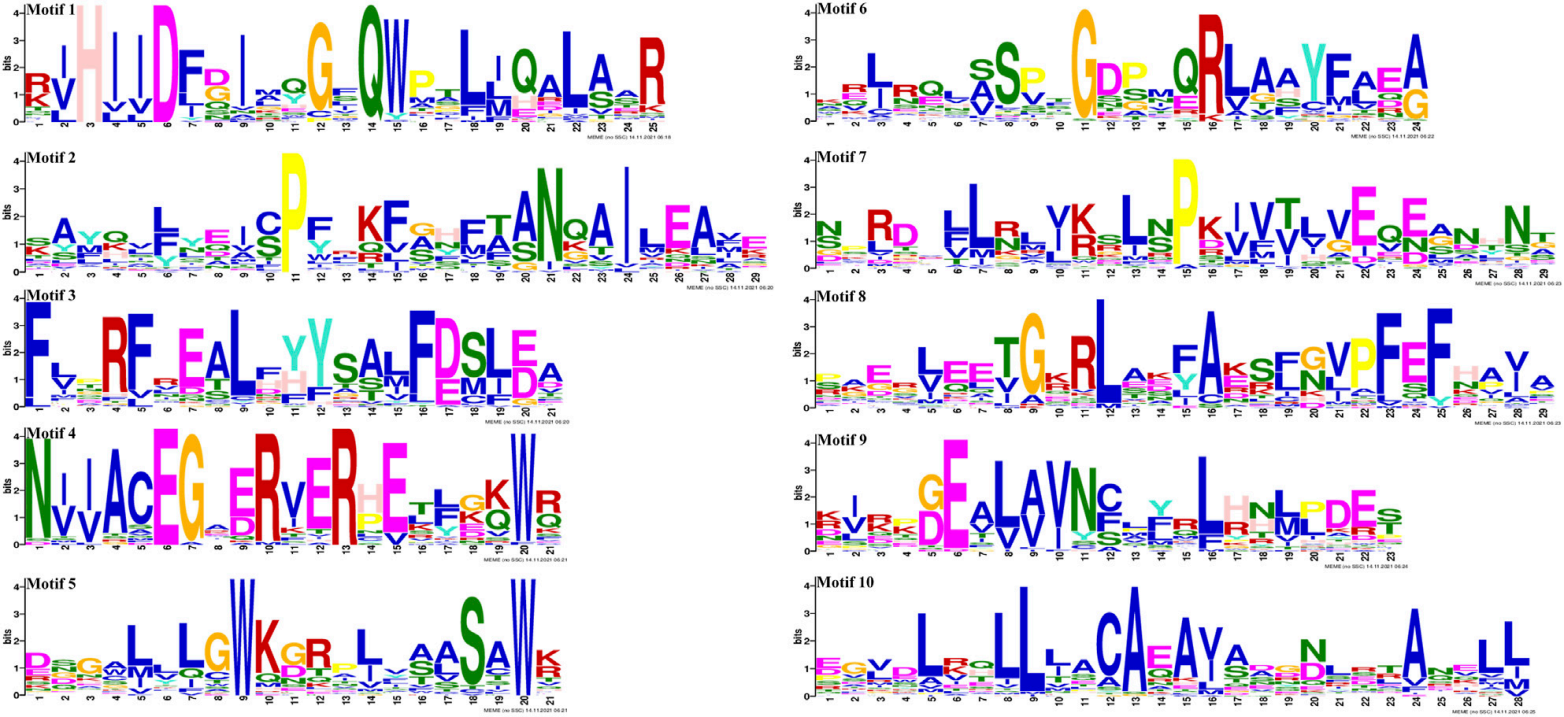
SmGRAS protein motifs confirmed using CDD of NCBI.

Gene structure analysis showed that up to 60.42% (29/48) of *SmGRASs* genes did not contain any introns (Figure 1B), and the members clustered in the same subfamily had similar gene structures (Figure 1). Motif analysis (Figure 1C) indicated that members grouped in the same subfamily possessed similar conserved motif composition and sorting order, suggesting that members clustered in the same subfamily might share similar functions. All members contained motifs 1, 2, 3, 5, 6, 7, and 8, indicating that these motifs played an important role in the *GRAS* gene family. Among them, motif 5 is found in the C terminus of all *SmGRAS*s, indicating that the C terminus of the *SmGRAS* gene family is relatively conserved, which is consistent with previous studies (Pysh et al., 1999). Using the CDD tool, a total of nine motifs (motifs 1–9) were functionally annotated for the components of the conserved GRAS domain. Moreover, conserved motif sequence analysis found that motif 1 contained a VHIID fragment in the GRAS domain, and motif 5 contained a SAW fragment in the GRAS domain (Figure 2).

### Phylogenetic analysis of GRAS proteins

**FIGURE 3:**
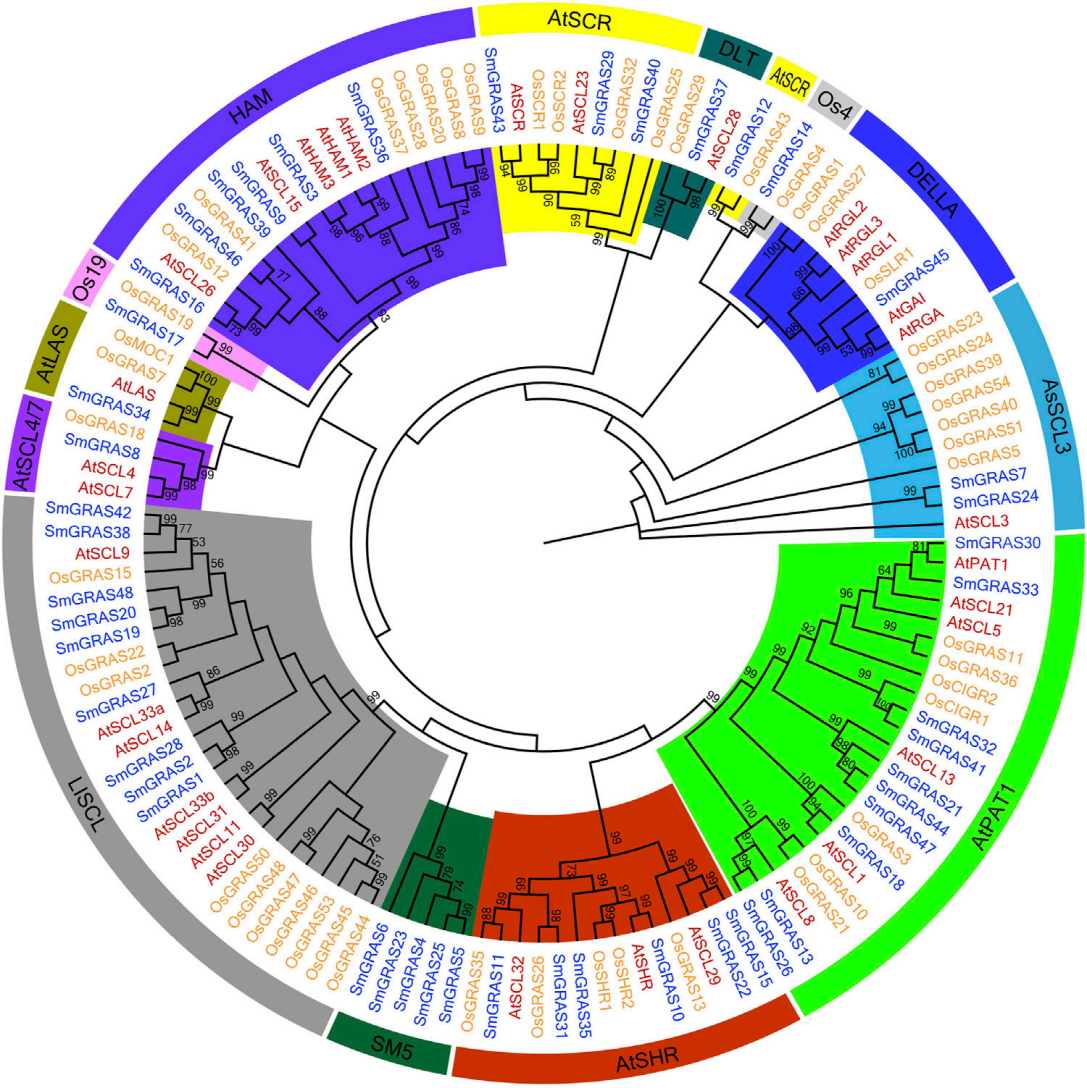
Combined phylogenetic analysis of GRAS proteins from *Arabidopsis,* rice, and eggplant. The rooted maximum likelihood tree was constructed from alignments of 131 GRAS protein sequences from *Arabidopsis* (33), rice (50), and eggplant (48) under the LG + G + F model with 1,000 bootstrap replications. Bootstrap values above 50% are shown. For each subfamily, bootstrap values are shown in different colors.

The phylogenetic trees of GRAS proteins of *A. thaliana* (33), rice (50), and eggplant (48) were constructed by using the MEGA-X tool (Figure 3). Upon consultation with previous systematic classification rules of *Arabidopsis* and rice GRAS subfamilies (Liu and Widmer, 2014), the GRAS proteins of *Arabidopsis*, rice, and eggplant were divided into 13 subfamilies. Each subfamily contained SmGRAS members, and the number of SmGRAS members in different subfamilies varied greatly. Among them, PAT1 had the largest number of SmGRAS members (10),followed by LISCL (9). In contrast, LAS, OS4, DLT, and OS19 had the smallest subfamily, which contained only one member. The majority subfamilies contained the common members across *A. thaliana*, rice, and eggplant. However, the SmGRAS5 was a new subfamily identified in this study, which only contained GRAS members in eggplant. Moreover, OS4 and OS19 of GRAS members were only identified in rice and eggplant, and no members of these families were found in *Arabidopsis*. These results indicated that the GRAS protein subfamily might undergo differentiation between monocotyledons and dicotyledons to some extent.

### Chromosomal location and collinearity analysis of SmGRASs

**FIGURE 4:**
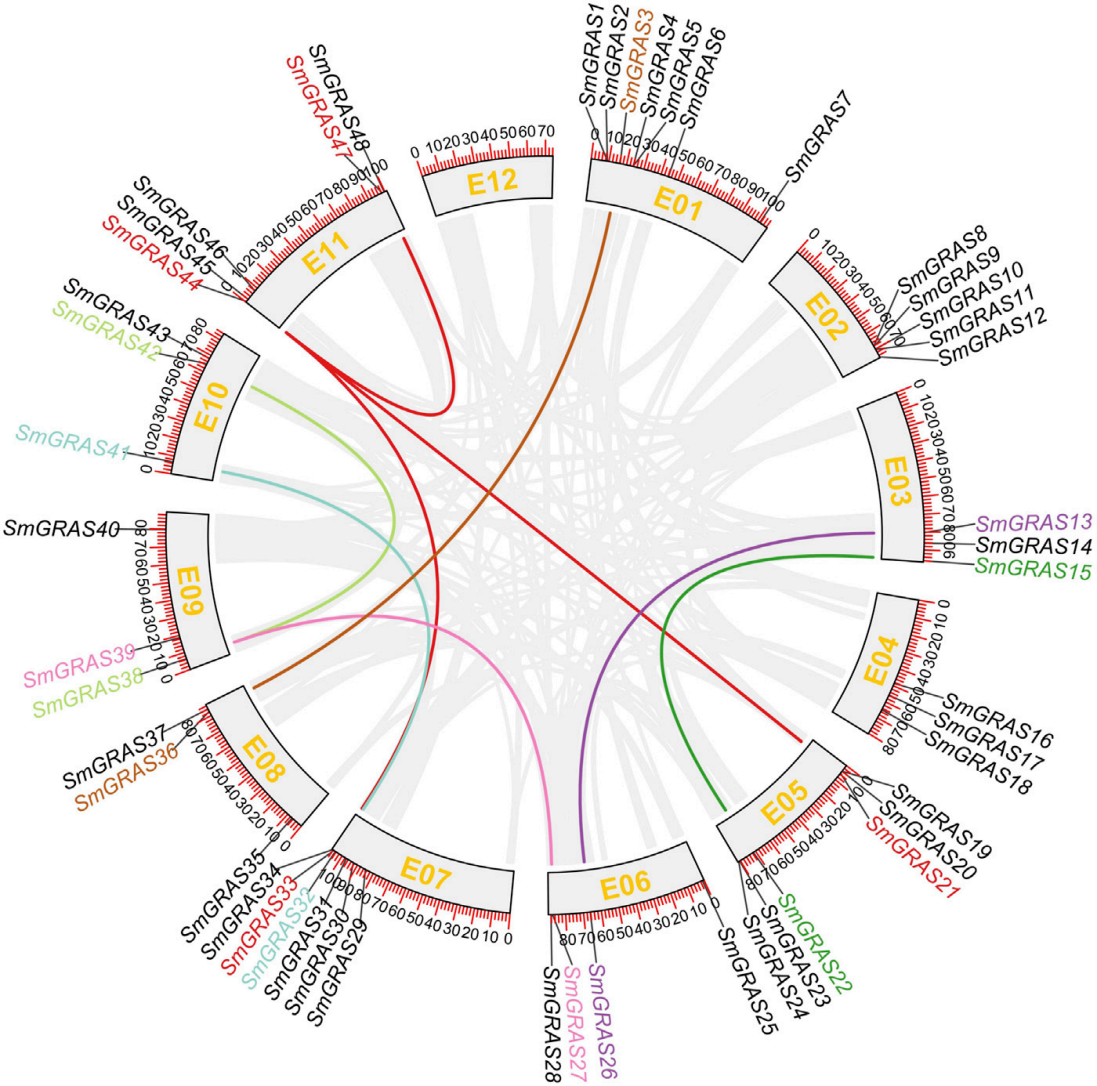
Collinearity analysis of SmGRASs. Scale bar on the chromosome shows the length of the chromosome (Mb). Genes with segmental duplication are shown in the same color.

**FIGURE 5:**
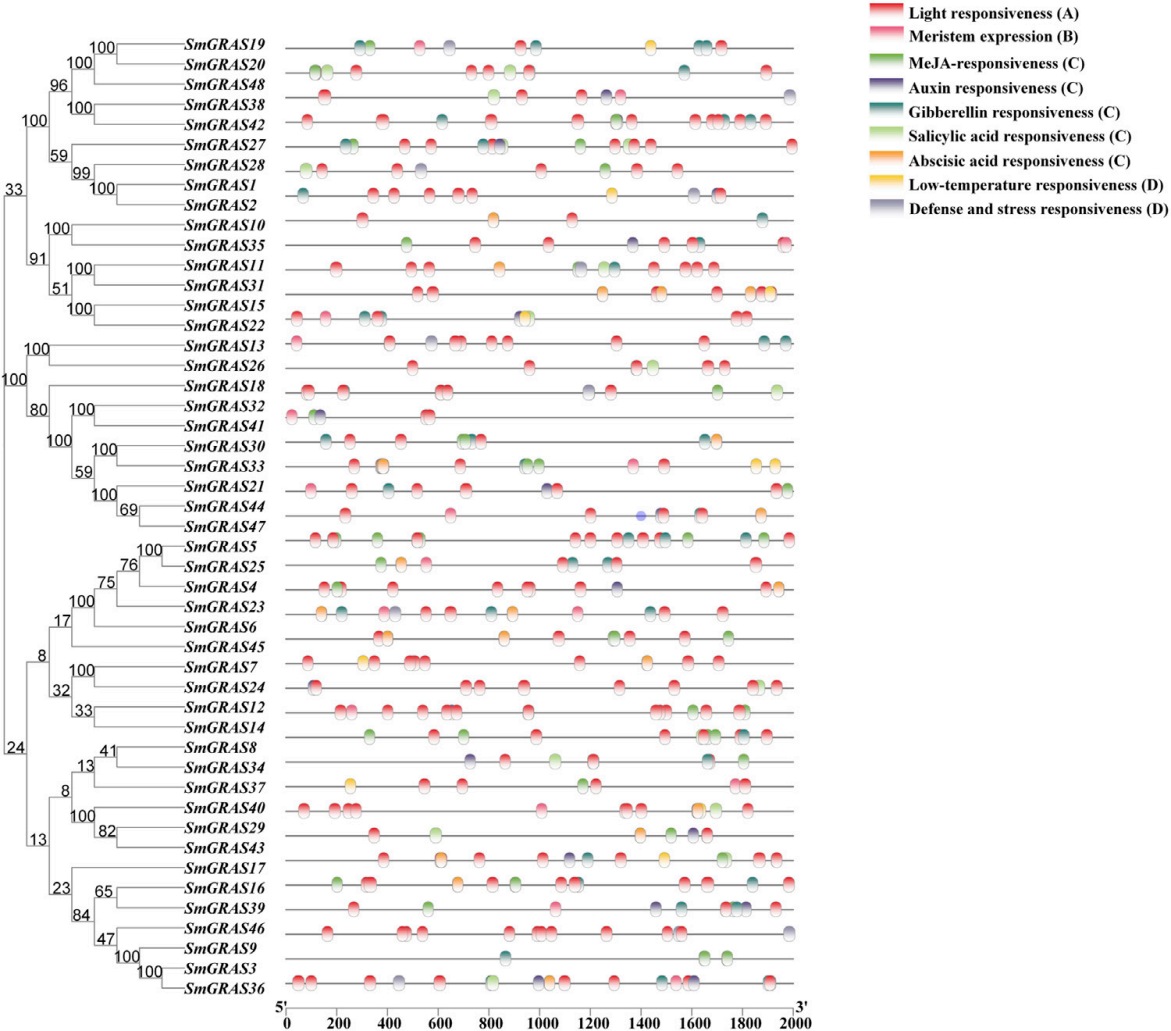
*Cis*-acting element analysis of *SmGRAS*s. Note: **(A)** Photoresponsive cis-acting element; **(B)** growth- and development-related cis-acting element; **(C)** hormone response-related cis-acting elements; **(D)** cis-acting elements associated with stress.

The 48 *SmGRASs* were unevenly distributed on 11 chromosomes of eggplant (Figure 4). The majority of *SmGRAS* members were distributed on chromosomes 1, 2, 5, 7, and 11, and chromosome 1 contained the largest number of *SmGRASs* (seven genes) while chromosomes 3, 4, 8, 9, and 10 all contained three SmGRAS members. Gene duplication events, including segmental duplication and tandem duplication (Cannon et al., 2004), were analyzed in this study. A total of nine *SmGRAS* gene pairs (3/36, 13/26, 15/2, 27/39, 32/41, 33/44, 30/42, 38/42, and 44/47) were identified (Figure 5). All of them were confirmed to be segmental duplication events.

### *Cis*-acting elements of *SmGRAS*s

The 2 kb upstream CDS sequences were extracted from the promoter regions of 48 *SmGRASs*. The *cis*-acting elements in the promoter region of *SmGRASs* were predicted by the PlantCARE online tool (Figure 5). Four types of cis-acting elements were found in the promoter regions of *SmGRASs*. Each *SmGRAS* gene contains 5–19 photoresponsive cis-acting elements, which is the most abundant type. This is followed by hormone-associated cis-acting elements consisting of a combination of abscisic acid, auxin, gibberellin, methyl jasmonate, and salicylic acid responsive elements, which all *SmGRAS* members contain. However, there were few cis-acting elements related to growth and development and stress response, and the *SmGRAS* members containing these two types of cis-acting elements were 18 (*SmGRAS04/08/12/15/16/18/19/22/25/30/33/35/36/37/40/41/44/48*) and 22 (*SmGRAS01/05/06/09/11/15/19/20/22/25/26/27/28/31/36/37/39/40/41/43/45/48*), respectively*.*

### Expression profiles of *SmGRAS*s

**FIGURE 6:**
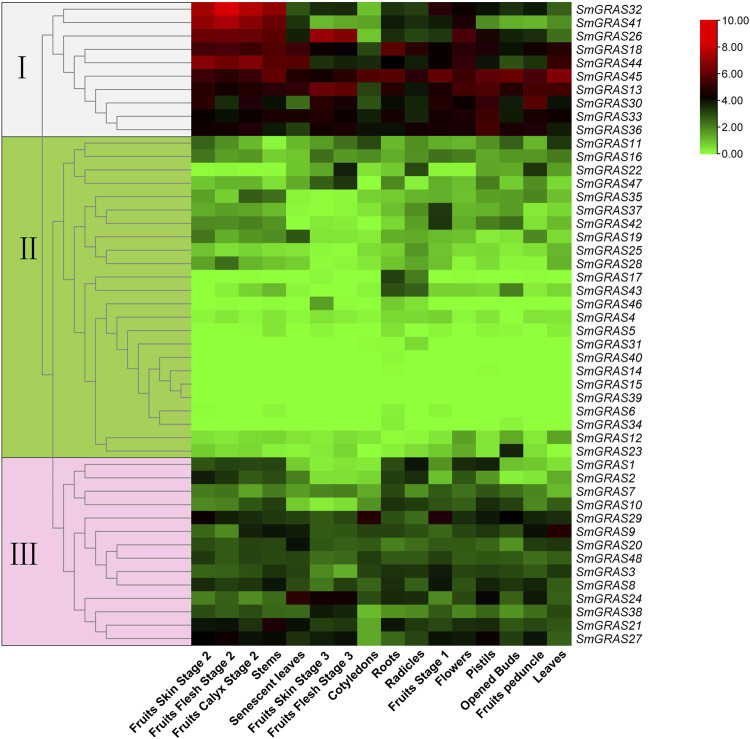
Expression analysis of Sm*GRAS*s in different tissues and different stages. The three colored boxes on the left of the picture symbol indicate the subfamily that the corresponding gene on the right belongs to.

To further explore the expression patterns of the *GRAS* genes, the transcript data of 16 tissues were obtained from the public genome database, including roots, stems, leaves, flowers, and fruits. A heatmap was generated based on the transcript data of 48 *SmGRAS* genes (Figure 6). As shown in Figure 6, some *SmGRAS*s exhibited distinct tissue-specific expression patterns, while others were expressed throughout in the whole plant. A total of 48 genes were grouped into 3 groups (Figure 6). Group Ⅰ contained 10 genes (*SmGRAS32/41/26/18/44/45/13/30/33/36*), which showed higher expression levels in the whole growth period, indicating that these genes might have a relatively important role in the whole growth stage of plants. In contrast, 24 genes in group II showed low or no expression in most tissues. A total of 14 genes (*SmGRAS1/2/7/10/29/9/20/48/3/8/24/38/21/27*) were included in group III, which were at moderate expression levels in most of the analyzed tissues.

### Expression of *SmGRAS*s in response to cold stress

**FIGURE 7:**
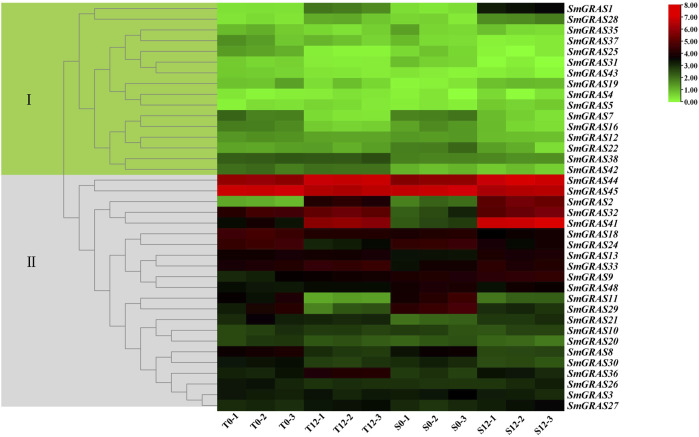
Expression changes of *SmGRAS*s under cold stress. S0 and T0 represent the sensitive and tolerant varieties before low-temperature treatment, respectively. S12 and T12 represent the sensitive and tolerant varieties after low-temperature treatment, respectively. This experiment was repeated three times. The genes on the right of the image correspond to the two color-coded boxes on the left.

To further investigate the expression patterns of *SmGRASs* in response to cold stress, the relative expression levels were measured based on the expression FPKM values (Figure 7). Under cold stress, the expression levels of different genes showed great variation, such as *SmGRAS2*, *SmGRAS7*, *SmGRAS8*, *SmGRAS16*, *SmGRAS24*, *SmGRAS29*, *SmGRAS32*, and *SmGRAS44*. Among them, *SmGRAS2*, *SmGRAS28*, *SmGRAS32*, *SmGRAS41*, and *SmGRAS44* showed significant upregulation, while the rest showed significant downregulation.

**FIGURE 8:**
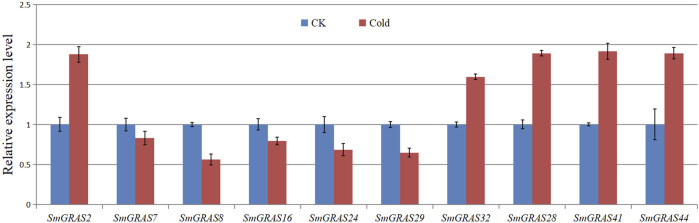
Relative expression levels of eight *SmGRAS*s analyzed using qPCR in response to cold after 12 h treatment compared with that of control (CK). The bars of expression with and without hypothermia treatment are shown in red and blue, respectively. *Y*-axis represents relative expression values, and *X*-axis represents different genes. Error bars are standard deviations of three biological replicates.

According to the transcriptome data (Figure 8), ten *SmGRAS* genes that exhibited significant change under cold stress were selected for further qRT-PCR analysis (Figure 8). The results showed that five genes (*SmGRAS2*, *SmGRAS28*, *SmGRAS32*, *SmGRAS41*, and *SmGRAS44*) were upregulated and five genes (*SmGRAS7/8/16/24/29*) were downregulated. A similar gene expression pattern was found between the qRT-PCR and transcriptome analysis (Figure 7).

## Discussion

In this study, a total of 48 *SmGRAS*s were identified from the eggplant genome, higher than that of *A. thaliana* (34) and lower than that of rice (60) and tomato(53) (Liu and Widmer, 2014; Huang et al., 2015). As we all know, the genome size of eggplant was 1.07 Gb (Wei et al., 2020), much higher than that of rice (420 Mb) (Goff et al., 2002), *A. thaliana* (125 Mb) (Kaul et al., 2000), and potato (844 Mb) (Diambra, 2011). Thus, it can be seen that *GRAS* gene families are widely distributed in plants, and the number of family members is independent of genome size (Xu et al., 2016), which may be due to the degree of genetic expansion between species. Collinearity analysis showed that nine *SmGRAS* gene pairs had collinearity relationships, and all of them were segmental duplication events, indicating that the amplification of eggplant *GRAS* protein was mainly dependent on segmental duplication. Meanwhile, these collinear genes were also grouped into the same group in evolutionary tree analysis, such as *SmGRAS21*, *SmGRAS33*, *SmGRAS44*,and *SmGRAS47*.

Phylogenetic tree, gene structure, and conserved motif analysis of *SmGRASs* showed that gene family members in the same group often had similar gene structures and conserved domains, but it was not excluded that some members had specificity. For example, in the HAM subgroup, only *SmGRAS36* contains an intron, which may be due to the evolution of genes within the group. In addition, the exon–intron analysis showed that 60.42% of *SmGRAS* genes were intron-less (Figure 1B), with proportions of 82,2%, 77,4%, 67.6%, 55%, and 54.7% in *P. mume,* tomato, *Arabidopsis*, rice, *and Populus* (Liu and Widmer, 2014; Huang et al., 2015; Lu et al., 2015), respectively. The high percentage of intron-less genes in the *GRAS* gene family in plants implies the close evolutionary relationship of GRAS proteins.

The functional studies on *GRAS* gene families have shown that they play an important role in various abiotic stress responses (Fan et al., 2017) and are extensively involved in stress resistance, signal transduction, and mycorrhizal formation in plants (Sun et al., 2012). This study analyzed the relative expression levels of *SmGRAS*s under low-temperature stress and found that the expression levels of 10 *SmGRASs* were significantly different after low-temperature stress compared with normal conditions. Among them, *SmGRAS2*, *SmGRAS32*, and *SmGRAS44* responded positively to low-temperature stress (Figure 8). Notably, *SmGRAS32* and *SmGRAS41* were segmentary replicate pairs (Figure 4), and their promoter regions both contained cis-acting elements related to cold stress (Figure 5), which was consistent with our qPCR results (Figure 8). The result suggested that segmentary replicate pairs may play similar functions. As we know, *SmGRAS32* and *SmGRAS44* belong to the PAT1 subfamily, indicating that members of this family can actively respond to low-temperature stress, which is consistent with the report of Yuan et al. (2016), and the genes included in this subfamily have potential applications in improving cold stress tolerance of plants. This study provides important information for further research on the function of GRAS transcription factors in eggplant under cold stress.

## Conclusion

In this study, a total of 48 *SmGRASs* were identified by genome-wide analysis, and then a comprehensive analysis of the identified *SmGRASs* was conducted. The structural diversity of *SmGRASs* may reflect their functional diversity. The collinearity analysis of *SmGRASs* found nine pairs of genes in collinear relationships, and they belonged to segmental duplication. The analysis of expression patterns of *SmGRASs* showed that these genes were expressed distinctly in different tissues of eggplant, and five genes (*SmGRAS2/32/28/41/44*) were positively responsive to cold stress. Our results provide vital information for the further exploration of the functional aspects of the gene family.

## Data Availability

The original contributions presented in the study are included in the article/Supplementary Materials; further inquiries can be directed to the corresponding authors.
