# Supplementary material for: Genome-wide identification and expression analysis of the GRAS transcription in eggplant (Solanum melongena L.)
Source: Front Genet. 2022 Sep 2;13:932731. doi: 10.3389/fgene.2022.932731 (PMC9478738; doi:10.3389/fgene.2022.932731)
Supplement: Supplementary file 1 [file Table1.docx]

Supplementary Material

**Supplementary Table S1.** qRT-PCR primers sequences used in this study

| **Primer name** | **Forward Sequence (5’ to 3’)** | **Reverse Sequence (5’ to 3’)** |
| --- | --- | --- |
| *SmGRAS2* | AGCAGAGAGGGTTGAGGACA | ATTCAGGACAGCATCCCTTG |
| *SmGRAS7* | TCACCATACCAATGGCTCAA | CCCATTCCAAGACCTCAAAA |
| *SmGRAS8* | CTGCACTCAATCTTGCCAAA | GTGAGTCTCGGGTCAAGCTC |
| *SmGRAS16* | TCAATGGGCTTCCTTAATGC | CTTCTCCGCGGACTAACTTG |
| *SmGRAS24* | GGTGAGGCTCTTGCGATTAG | GAACATAGCGGGGATGAAGA |
| *SmGRAS28* | TGAATCACCAAGGGTTCACA | CTCTCAGCTGGTCGAAAACC |
| *SmGRAS29* | GGGTGAAAATTGGGGAAACT | ACGAACCGCCCTAAAAACTT |
| *SmGRAS32* | GCCAGTTTTTGCTCCTGAAG | TTGTTCCACCAAAGTGACCA |
| *SmGRAS41* | CTCTCAGCTGGTCGAAAACC | TTCACGTCAACGCTCTCATC |
| *SmGRAS44* | CCTTCCAAGATTCCGTGAAA | CATTTGGCATGGGGTAAATC |
| *SmActin* | GTCGGAATGGGACAGAAGGATG | GTGCCTCAGTCAGGAGAACAGGGT |
